# CMIP interacts with WT1 and targets it on the proteasome degradation pathway

**DOI:** 10.1002/ctm2.460

**Published:** 2021-07-22

**Authors:** Shao‐Yu Zhang, Qingfeng Fan, Anissa Moktefi, Virginie Ory, Vincent Audard, Andre Pawlak, Mario Ollero, Dil Sahali, Carole Henique

**Affiliations:** ^1^ INSERM Creteil France; ^2^ Faculté de santé Université Paris Est Creteil Creteil France; ^3^ AP‐HP Groupe hospitalier Henri Mondor‐Albert Chenevier Département de pathologie Creteil France; ^4^ AP‐HP Groupe Henri‐Mondor Albert‐Chenevier Service de Néphrologie Creteil France

**Keywords:** CMIP, gene therapy, nephrotic syndrome, podocyte, WT1

## Abstract

**Background:**

The Wilms tumor 1 suppressor gene, *WT1*, is expressed throughout life in podocytes and is essential for their function. Downregulation of WT1 has been reported in podocyte diseases but the underlying mechanisms remain unclear. Podocyte injury is the hallmark of idiopathic nephrotic syndrome (INS), the most frequent glomerular disease in children and young adults. An increase in the abundance of Cmaf‐inducing protein (CMIP) has been found to alter podocyte function, but it is not known whether CMIP affects WT1 expression.

**Methods:**

Transcriptional and post‐transcriptional regulation of WT1in the presence of CMIP was studied using transient transfection, mouse models, and siRNA handling.

**Results:**

We showed that overproduction of CMIP in the podocyte was consistently associated with a downregulation of WT1 according to two mechanisms. We found that CMIP prevented the NF‐kB‐mediated transcriptional activation of WT1. We demonstrated that CMIP interacts directly with WT1 through its leucine‐rich repeat domain. Overexpression of CMIP in the M15 cell line induced a downregulation of WT1, which was prevented by lactacystin, a potent proteasome inhibitor. We showed that CMIP exhibits an E3 ligase activity and targets WT1 to proteasome degradation. Intravenous injection of Cmip‐siRNA specifically prevented the repression of Wt1 in lipopolysaccharides‐induced proteinuria in mice.

**Conclusions:**

These data suggest that CMIP is a repressor of WT1 and might be a critical player in the pathophysiology of some podocyte diseases. Because WT1 is required for podocyte integrity, CMIP could be considered a therapeutic target in podocyte diseases.

## Graphical abstract

- INS patients show increased CMIP and decreased WT1 expression in podocytes.
- CMIP inhibits NF‐κB‐driven WT1 transcription in M15 cells.
- CMIP targets WT1 to proteasome degradation via ubiquitin ligase activity in M15 cells.
- RNAi against Cmip restores Wt1 expression in a mouse model of proteinuria.

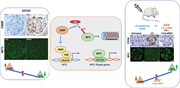

**ABBREVIATIONS**
AP‐1: activator protein‐1
BSA: bovine serum albumin
CMIP: cmaf‐inducing protein
CRE: cAMP response element
DAPK: death‐associated protein kinase
DIP‐1: DAPK‐interacting protein‐1
DMSO: dimethylsulfoxyde
DSP: dithiobis‐succinimidylpropionate
Erk: extracellular signal‐regulated kinases
FSGS: focal and segmental glomerulosclerosis
GHSR: growth hormone secretagogue receptor (also called Ghrelin receptor)
GST: glutathione‐S‐transferase
HA: hemagglutinin
HEK: human embryonic kidney
HEPES: hydroxyethyl‐piperazineethanesulfonic acid
hRL: humanized renilla luciferase
INS: idiopathic nephrotic syndrome
KCl: potassium chloride
KTS: lysine, threonine and serine
LPS: lipopolysaccharides
LRR: leucine‐rich repeat
M15: murine embryonic mesonephros‐derived cell line
Mab: monoclonal antibody
MCNS: minimal change nephrotic syndrome
MgCl: magnesium chloride
miRNA: micro ribonucleic acid
mRNP: messenger ribonucleoprotein
NaCl: sodium chloride
NF‐κB: nuclear factor‐κB
Pax2: paired box gene 2
PBS: phosphate‐buffered saline
Pc Ab: polyclonal antibody
PH: pleckstrin homology
PI3K: phosphatidylinositol 3‐kinase
PKC: protein kinase C
RNA: ribonucleic acid
RT‐PCR: realtime PCR
SDS: sodium dodecyl sulfate
SH: Src homology
siRNA: small interfering RNA
Tris‐HCl: tris(hydroxymethyl)aminomethane‐hydrochloride
Ub: ubiquitin
Wnt: wingless/integrated
WT1: Wilms tumor 1
ZnCl: zinc chloride

## 1 BACKGROUND

The pathogenesis of human diseases relies commonly on a disorder of gene regulation, which is a complex process involving a balance between many modulators, such as transcription activators or repressors, and factors acting in both cytoplasm and nuclear compartments. A growing list of proteins involved in gene expression and regulation appear to be multifunctional, acting at different cellular structures through their interacting domains or multiple docking sites. Besides proteins, microRNAs (miRNA) are found as other regulators of gene expression and participate in many biological processes. Although intense efforts have been pursued to clarify the pathogenesis of podocyte diseases, we are still far behind from elucidating the underlying molecular mechanisms.

Podocyte is considered as a target in many systemic or primary glomerular diseases. However, recent clinical and experimental evidences point out also the role of podocyte as an actor in the pathogenesis of glomerular diseases. Podocyte injury triggers several molecular events such as alteration of transcription machinery resulting in podocyte dedifferentiation and downregulation of proteins playing a key role in their functional integrity. Remarkably, podocyte injury alters cell programing and leads to induction of proteins, normally silenced, that initiate or contribute to cell damage and podocyte loss, including neighboring healthy podocytes.^1^ For instance, Wnt and Pax2 are highly expressed in kidney development, become silenced in adult kidney but are reactivated in some types of glomerular diseases.^2^, ^3^

Wilms tumor 1 (*WT1*) gene encodes a zinc finger transcription factor (WT1) that regulates genes involved in proliferation, differentiation and apoptosis, while it plays a central role in the embryogenesis of many tissues including the urogenital system, heart, and central nervous system.^4^ Human WT1 plays multiple roles at several stages of nephrogenesis. In metanephric mesenchyme, WT1 activates FGF, represses Smad signaling, and inhibits Wnt signaling by activating CXXC5, a Wnt/β‐catenin inhibitor.^5^ In addition, WT1 binds and activates Fgf8 and Wnt4, two transcription factors that are required for mesenchymal‐epithelial transition leading to formation of renal vesicle.^6^ In adult kidney, the expression of WT1 is restricted to the podocyte, where it plays a central role in differentiation, structure, function, and maintenance throughout adult life. Indeed, mouse Wt1 activates podocalyxin, nephrin, Bcl‐2, Gas1, Sciellin and Sulfatase1, while it represses TGFβ1, EGF receptor, and PDGF‐A, among others.^7^, ^8^, ^9^, ^10^, ^11^, ^12^, ^13^

Although predominantly expressed in nucleus in normal cells, WT1 shuttles between the nuclear and cytoplasmic compartments.^14^ WT1 is detected in functional polysomes; interacts with the splice factor U2AF65; and is associated with nuclear mRNP particles in fetal kidney cells.^15^, ^16^ In WT1‐expressing tumors, WT1 is mostly expressed in the cytoplasm^17^ and interacts with the actin cytoskeleton.^18^

In acquired human nephrotic syndromes, low WT1 expression at the mRNA and protein level has been reported, with a shift from nuclear to cytoplasm compartment.^19^ It has been shown that the abundance of Wt1 was reduced in a reversible fashion in experimental nephrotic proteinuria induced by lipopolysaccharides (LPS)^20^ and adriamycin.^21^ However, the factors that regulate the expression and cellular segregation of WT1 are little known.

Cmaf‐inducing protein (CMIP) is a newly identified gene that encodes an 86 kDa protein, containing an N‐terminal pleckstrin homology domain (PH), a nuclear localization signal near the PH domain, a middle region characterized by the presence of several interacting docking sites, including a 14‐3‐3 module, a PKC domain, an Erk domain, an SH3 domain similar to the p85 regulatory subunit of phosphatidylinositol 3‐kinase (PI3K), and a C‐terminal leucine‐rich repeat (LRR) domain.^22^ Because no DNA binding domain has been identified in the primary structure, it is assumed that CMIP is not a transcriptional factor. The nucleotide sequences of human (CMIP) and mouse Cmip coding regions are highly homologous (91%), while the encoded proteins are nearly identical (98.3%). Several investigations using different approaches have shown that CMIP is a newly‐recognized adapter, multifunctional protein interfering with multiple signaling pathways through protein‐protein interaction with multiple partners including Src kinase Fyn, PI3 kinase (p85), cytoskeletal protein filamin‐A, transcription factor NF‐kB (RelA), death‐associated protein kinase (DAPK)‐interacting protein‐1 (DIP‐1).^23^, ^24^, ^25^, ^26^, ^27^ We report here several lines of evidence suggesting that CMIP exerts *in vitro* and *in vivo* a downregulation of WT1 at the mRNA and protein levels.

## 2 METHODS

### 2.1 Patients

The patients included in this study were evaluated in our nephrology department for nephrotic syndrome. All patients had proteinuria above 3 g/24 h and hypo‐albuminemia (<30 gr/L) at the time of the kidney biopsy, which was performed before the initiation of steroids or immunosuppressive therapy. Minimal change nephrotic syndrome (MCNS) and focal and segmental glomerulosclerosis (FSGS) were clinically classified as idiopathic in all cases. Samples were obtained in the context of a clinical trial (Clinical trial.gouv identifier: NCT01197040) with written consent of participants involved in this study. Control kidney samples were supplied by the hospital tissue bank (platform of biological resources, Henri Mondor hospital) from patients undergoing nephrectomy for polar kidney tumor.

### 2.2 Plasmid constructs, cell culture, and transfections

The *WT1* expression plasmids are a kind gift from Dr Andreas Schedl (INSERM U636, Nice, France). The human *WT1* promoter constructs have been previously described^28^, ^29^ and are a kind gift from Dr Michael Eccles (Department of Biochemistry, University of Otago, Dunedin, New Zealand). The *AP‐1*‐luciferase and *pCRE*‐luciferase reporter plasmids were purchased from Promega. A phRL‐null vector, containing a renilla luciferase gene (Promega), was used as an internal control for transfection. The *NF‐κB p50* and *p65* expression vectors have also been described.^30^ The *HA‐ubiquitin* plasmid is a kind gift from Dr Gratton (Laboratory of Endothelial Cell Biology, Institut de recherche clinique de Montreal, Canada). The human *CMIP* constructs have been previously described.^23^ Like human *CMIP* mRNA (accession number: NM_198390), the coding sequence of mouse *Cmip* transcript (accession number: XM_001004724) contains 2322 nucleotides, which encodes a protein of 773 amino acid residues with an expected size of 83 kDa. Sequence comparison analysis indicates that human and mouse Cmip share 98.4% homology. The mouse full‐length *Cmip* mRNA was prepared from mouse glomerular extracts using the gateway system with the oligonucleotides listed in Table 1, as previously described for human *CMIP* transcript.^23^

**TABLE 1:** Sequence of primers and PCR conditions

Primers	Sequences	Accession number	Expected size	Annealing temperature (°C)
Mouse WT1 transcript Mouse full‐length Cmip Mouse 18S Human CMIP N (PH) Human CMIP C (LRR) Mouse Cmip (PH)	Forward: 5′‐ACCCAGGCTGCAATAAGAG‐3′ Reverse: 5′‐CAGCTGGAGTTTGGTCATGT‐3′ Forward: 5′‐GGGGACAAGTTTGTACAAAAAAGCAGGCTTCGAA GGAGATAGAACCATGGATGTGACGAGCAGCTCGG GCGGCGGCGACCCC‐3′ Reverse: 5′‐GGGGACCACTTTGTACAAGAAAGCTGGGTCCCA GGCTTCCGTGTAGCGAACATCAACCTCCT‐3′ Forward: 5′‐GTAACCCGTTGAACCCCATT‐3′ Reverse: 5′‐CCATCCAATCGGTAGTAGCG‐3′ Forward: 5′‐ATGGATGTGACCAGCAGCTCGGGCGGCGGCGGC GAC‐3′ Reverse: 5′‐GTTCCATTCTCTGCAATGGAAGAAAAAGATT‐3′ Forward: 5′‐GAGTTCATCAACAGCCGCGACAATTCC‐3′ Reverse: 5′‐GAAGTGGACGTCCGCTACACCGAAGCCTGG‐3′ Forward: 5′‐ATGGATGTGACGAGCAGCTCGGGCGGCGGCGAC‐3′ CCC Reverse: 5′‐ACGCAGAGGTAAGGTGGGCTTTCGGTCGC‐3′	NM_144783 XM_001004724.2 NR_003278 NM_198390.2 NM_198390.2 XM_001004724.2	362 2321 50 486 1272 1125	57 62°C 55 62 62 60

The underlined sequences correspond to attB‐sites used to incorporate the PCR product into the gateway plasmids.

Cultures of human embryonic kidney cells (HEK 293) and immortalized mouse podocytes have been performed as previously described.^23^ Murine embryonic mesonephros‐derived M15 cell line has been described elsewhere.^31^ In some experiments, HEK cells were incubated 4 h post‐transfection with 2 μM of lactacystin (Sigma‐Aldrich; St Louis, MO) for various times (16, 20 and 32 h). Protein extraction was performed at a later time, as indicated in the text.

### 2.3 Generation of CMIP transgenic mice

The generation of Cmip transgenic mice has been previously described.^23^ Briefly, transgenic mice were obtained using a targeting system based on the reconstitution of a functional X‐linked HPRT locus (that is lacking in the parent embryonic stem cells) by homologous recombination, such that only properly integrated ES cells survive to HAT selection.^32^ Three plasmids were used to construct the Hprt targeting vectors. The first plasmid comprises an 8.3‐kbp fragment of the murine promoter and the 5′‐untranslated sequence of the nephrin gene.^33^ The full‐length coding sequence of Cmip was inserted into the XhoI site, downstream from the nephrin segment. A 13.275 kbp‐fragment containing the transgene (nephrin segment and Cmip) was excised by digestion with *Nar*I and *Pvu*I restriction enzymes, then inserted into the pEntr1A gateway vector, using the Quick ligase (New England Biolabs, France). The transgene was subsequently subcloned by homologous recombination into the pDest vector, upstream of the promoter, and the exon 1 of the human Hprt. The recombinant clones were confirmed by *Bam*HI, *Eco*RV, and *Hind*II restriction analysis. The resulting plasmid was micro‐injected into BPES (hybrid c57BL/6 and 129) ES cells. Homologous recombinants selected on HAT‐supplemented medium HAT‐resistant clones were confirmed by PCR and expanded for 10 days. Targeted BPES (hybrid C57BL/6/129) cells were injected into blastocytes. The BPES cells lead to an enhanced ES lineage contribution in chimeras and ensure 100% germ line transmission. Male chimeras with 100% brown coat color were bred to wild type (Wt) C57BL/6 females to obtain agouti offspring. Female agouti offspring were backcrossed with Wt C57BL/6 males to obtain hemizygous male mice. Successive backcrosses were performed in order to obtain a homogeneous C57BL/6 genetic background (≥ tenth generation). The experimental procedures were approved by the National Ethical Committee (ComEth), under accreditation number 201704061048410

### 2.4 Immunohistochemistry and confocal microscopy analyses

Primary antibodies used in this study included polyclonal antibodies, rabbit anti‐WT1 (C19, Santa Cruz Biotechnology, CA), guinea pig anti‐nephrin (Progen, Heidelberg, Germany). The CMIP polyclonal antibody was produced in rabbits immunized with peptides raised against peptides located in PH and LRR.

Immunofluorescence on kidney sections was performed on 4‐μm thick cryostat sections fixed in acetone for 10 min, air‐dried for 30 min at room temperature, then incubated in PBS for 3 min, and blocked in 1% BSA‐PBS. The sections were incubated with the indicated antibodies for 1 h at room temperature, washed with PBS, and incubated with FITC or red‐conjugated secondary antibodies. After washing with PBS, the slides were simultaneously incubated with FITC‐conjugated goat anti rabbit IgG and alexia‐555 goat anti‐guinea pig IgG. Tissue sections were imaged by a confocal laser scanning microscope LSM510‐META (Carl Zeiss, Germany) using a Plan‐Apochromat 63 X, 1.4 numerical aperture oil immersion objective. Acquisitions were performed with an argon laser (excitation wavelength 488 nm), and the emission of fluorescence was collected with the META channel between 500 and 600 nm. The pinhole was set at 1.0 Airy unit (0.8 mm optical slice thickness). The images were processed with ImageJ software (http://rsb.info.nih.gov/ij/). For CMIP quantification, we used a high cutoff value for the lower threshold to eliminate nonspecific signals. The lower and upper thresholds of fluorescence intensity (F) were fixed at 2000 and 4095 pixels, respectively. The area of specific labeling (lining the capillary loops) was normalized with respect to total glomerular area (S = labeled area/total area). The semi‐quantification (Q) of site‐specific fluorescent labeling was determined as follows: Q = F x S.

For immunohistochemistry study, antigen retrieval was performed by immersing the slides in boiling 0.01 M citrate buffer in a 500 W microwave oven for 15 min. Slides were incubated with the blocking reagents of avidin solution for 15 minutes and after washing with TBS buffer, slides were then treated with blocking reagent of biotin for another 15 minutes and normal blocking serum for 60 minutes. The slides were incubated overnight with anti‐CMIP or anti‐WT1 antibody (both polyclonal from rabbit), then with biotinylated secondary antibody. Before development, the endogenous peroxidase activity was blocked with 1% H2O2 in methanol for 10 min. An avidin‐biotinylated horseradish peroxidase complex (Vectastain ABC Reagent, Vector Laboratories; Burlingame, CA) and 3,3′‐diaminobenzidine (Sigma‐Aldrich; St Louis, MO) as a chromogen were applied for visualization of the immunoreaction. Omission of the primary antibody was considered as a negative control.

### 2.5 Luciferase reporter assays

The effect of *CMIP* on *WT1* transcriptional activity was determined by dual luciferase reporter assays driven by *WT1* promoter. HEK cells were co‐transfected with *WT1*‐luciferase reporter plasmid and CMIP with/without NF‐kB expression plasmids. The c‐fos expression plasmid was a gift from Dr Tom Curran.^34^ The Ghrelin receptor (GHSR [growth hormone secretagogue receptor]) was a gift from Dr Serge Amselem.^35^ The total amount of DNA was kept constant by replacing an *expression* plasmid by empty pcDNA vector. Series of transfections were performed in 500 μl‐wells. Twenty‐four hours after transfection, cells were lysed, and luciferase activity was measured in duplicate on a synergy HT spectrophotometer (Bio Tek, Winooski, VT, USA) using Dual‐Luciferase Reporter Assay System (Promega), as previously described.^36^ The relative luciferase activities were normalized to protein concentration of lysates determined by Bradford reaction assay (Sigma‐Aldrich, France).

### 2.6 Quantitative real‐time reverse transcription polymerase chain reaction

Kidney fractions enriched in glomeruli were isolated by sequential sieving. Total RNA was isolated using an RNeasy kit (Qiagen, Chatsworth, CA). The mouse Wt1 primers (Forward: ACCCAGGCTGCAATAAGAG; Reverse: CAGCTGGAGTTTGGTCATGT) amplified a 362‐bp sequence. Quantitative PCR (qPCR) was performed using SYBR green and the Light Cycler according to the manufacturer's instructions (Roche Diagnostic, France). Quantification was performed by the 2^ΔΔCt^ method using ribosomal 18S RNA normalization, and results were expressed as fold induction over values obtained from wild controls.

### 2.7 Western blots and immunoprecipitation analyses

The primary antibodies used in this study included anti‐WT1 (polyclonal: c‐19 and monoclonal: F6, Santa Cruz Biotechnology), anti‐NF‐kB p50 (Santa Cruz Biotechnology), anti‐GST (cell signaling), anti‐HA‐Tag (cell signaling), and anti‐V5 (Invitrogen). Control IgGs were purchased from Alpha Diagnostic International (San Antonio, Texas, USA).

For the preparation of mouse glomerular extract, fractions of kidney cortex were enriched in glomeruli by successive sieving through 105‐, 75‐, and 40‐μm cell strainers, as previously reported.^23^ Glomerular protein extracts were prepared in lysis buffer (50 mM Tris HCl pH 7.5, 150 mM NaCl, 2 mM EDTA, 1% NP40, 0.5% sodium deoxycholate, 0.1% SDS, 1 mM PMSF, 1 mM protease inhibitors, 1 mM NaF and 1 mM sodium orthovanadate). The protein lysates were resolved by SDS‐PAGE and analyzed by Western blot with the indicated antibodies.

### 2.8 In vivo cross‐linking

M15 cells were transfected with mouse Cmip expression plasmid and, 24 h later, were treated for 20 min with 0.5 mM cross‐linker dithiobis‐succinimidylpropionate (DSP) (Sigma‐Aldrich, France) or corresponding dilution of DMSO, as described previously.^18^ Cell protein extracts were prepared in lysis buffer (10 mM Tris‐HCl pH 7.4, 10 mM NaCl, 0.4% NP‐40, 3 mM MgCl_2_ and 1 mM of protease inhibitor cocktail) with or without 8 M urea. Three hundred microgram protein extractions were diluted 1:10 in immunoprecipitation (IP) buffer (20 mM HEPES pH 7.5, 70 mM KCl, 5 mM MgCl_2_, 0.05% NP‐40, 10% glycerol, 0.1 mM ZnCl_2_, 1 mg/ml BSA and 1 mM of protease inhibitor cocktail), in one ml final volume, and used for immunoprecipitation with rabbit anti‐WT1 antibody (C‐19, Santa Cruz) or nonimmune rabbit IgG as negative control (Alpha Diagnostic International, San Antonio, Texas, USA). After extensive washes in RIPA buffer (20 mM Tris pH7.6, 120 mM NaCl, 0.01% SDS, 0.01% deoxycholate, 0.4% NP‐40 and 1 mM of protease inhibitor cocktail), Protein‐A Sepharose precipitates were cleaved by boiling for 5 min. The samples were analyzed by Western blot using mouse anti‐WT1 (F‐6, Santa Cruz) and rabbit anti‐CMIP antibodies.

### 2.9 E3 ligase activity assay

To test E3 ligase activity of CMIP, we used the E_3_ ubiquitin ligase kit, according to the manufacturer's protocol (LifeSensors, Malvern, PA). The assay is based on the detection of polyubiquitin chains formed in an E3 ligase‐dependent reaction and captured by anti‐polyubiquitin reagent precoated in microtiter plate wells. CMIP was immunoprecipitated from HEK cells transfected with the corresponding expression plasmid (pDEST27‐CMIP) by using GST‐agarose beads (25 μl per reaction). Following purification, GST‐agarose beads were extensively washed and resuspended in 2X assay buffer (100 mM Tris‐HCl pH 8, 10 mM MgCl_2_, 2 mM β‐Mercaptoethanol). The reaction was conducted in 100 μl final volume by precoated well, containing 25 μl of enzyme solution (5 nM E1 activating enzyme and 100 nM E2 conjugating enzyme), 20 μg ubiquitin and 25 μl of serial dilution CMIP. Preliminary experiments have shown that Ubc13, an E2 conjugating enzyme, works well with CMIP. The enzymatic reaction was started following addition of 50 μl of 0.4 mM ATP and incubated 1 h at room temperature. The reaction volume was then discarded, each well was washed three times with phosphate buffered saline containing 0.1% Tween‐20 (PBST) and 5% BSA and incubated with 100 μl of detection solution for 1 h at room temperature. After extensive washing, each well was incubated with 100 μl of streptavidin‐HRP (dilution 1/10000 in PBST 5% BSA) for 1 h. After washing, 100 μl of Enhanced Chemiluminescent reagent (ECL, Thermofisher scientific, France) was added for each well for 5 min, then the relative luminescence unit (RLU) was measured using a Fusion‐SL Quick start imaging system (Vilbert Loumat SAS, France). For the control of the specificity of the reaction, we transfected HEK cells with the expression plasmids encoding the CMIP‐PH domain or the NF‐kB p50 subunit, then we performed immunoprecipitation from respective protein lysates as indicated above. The blank control includes all reagents (E1‐E2/Ubc13‐ubiquitin cocktail) minus the E3 ligase, whereas the positive control was provided in the kit and includes an E3 ligase. The generation of polyubiquitin chains was trigged in the presence of 0.2 mM ATP and detected by enhanced chemiluminescent reaction (ECL). Duplicate wells were used, and all values measured minus those obtained in the blank control in each dilution were represented in the graph.

### 2.10 In vitro activity and stability assays of stealth RNAis

We selected three sequences located in the open reading frame and that are conserved between human, rat, and mouse to be tested *in vitro*. The RNAi sequences are G6 (forward strand: UCCUGCUAUGAAGAGUUCAUCAACA), G8 (forward strand: CGGACCUUUCUCAGCAAGAUCCUCA), and G10 (forward strand: AAGAGUUCAUCAACAGCCGCGACAA). Stealth RNAis were synthesized by Invitrogen (Invitrogen, CA). The sense strand is inactivated using chemical modifications, which prevent its loading into the RISC complex and cannot induce off target effects. To avoid a microRNA effect (siRNA binding the 3′UTR region and acting on translation), the seed region was used in a Smith waterman alignment analysis against human, mouse, and rat coding regions (www.invitrogen.com/rnaidesigner).

The *in vitro* activity of Stealth RNAis was determined in HEK cells as previously described.^23^ The maximum inhibition (>85%) was obtained with the stealth RNAi 'G8', which was used for *in vivo* experiments.

### 2.11 siRNA treatment

Male BALB/c mice, 6–8 weeks of age and weighing 20−22 g, were purchased from Charles River Laboratory (France). Alexa Fluor647‐labeled Stealth Cmip siRNA (10 mg/kg) was mixed with Invivofectamine (ratio: 1/1, w/v), according to the manufacturer's instructions (Invitrogen, CA, USA), and the Invivofectamine‐Cmip siRNA complex (100 μl final volume) was injected into the internal jugular vein of mice (*n* = 10). Thirty minutes after siRNA injection, LPS (200 μg in 200 μl final volume) was injected intraperitoneally. Control mice were injected with an equal amount of either LPS (*n* = 5) or Invivofectamine alone (*n* = 5). Mice were kept in cages. Twenty‐four hours later, urine was collected, mice were sacrificed, and kidneys were harvested and processed for immunohistochemistry analysis. The efficiency of siRNA delivery was determined by immunofluorescence analysis on kidney cryosections fixed in formalin. The presence of Cmip was analyzed by immunohistochemistry. Double immunostaining was performed with WT1 (C‐19) and nephrin (Progen, Heidelberg, Germany) antibodies and analyzed by confocal microscopy.

### 2.12 Statistical analysis

The data presented are means ± SD and were prepared with GraphPad Prism software, version 8.0 for Macintosh (GraphPad Software, Inc, USA). A one‐way ANOVA test was used for comparison of multiple groups, while unpaired Student's or Mann Whitney tests were used as indicated in the figure legends. A *p*‐value of less than 0.05 was considered significant.

## 3 RESULTS

### 3.1 Increased CMIP abundance is associated with WT1 downregulation in acquired podocyte diseases

**FIGURE 1:**
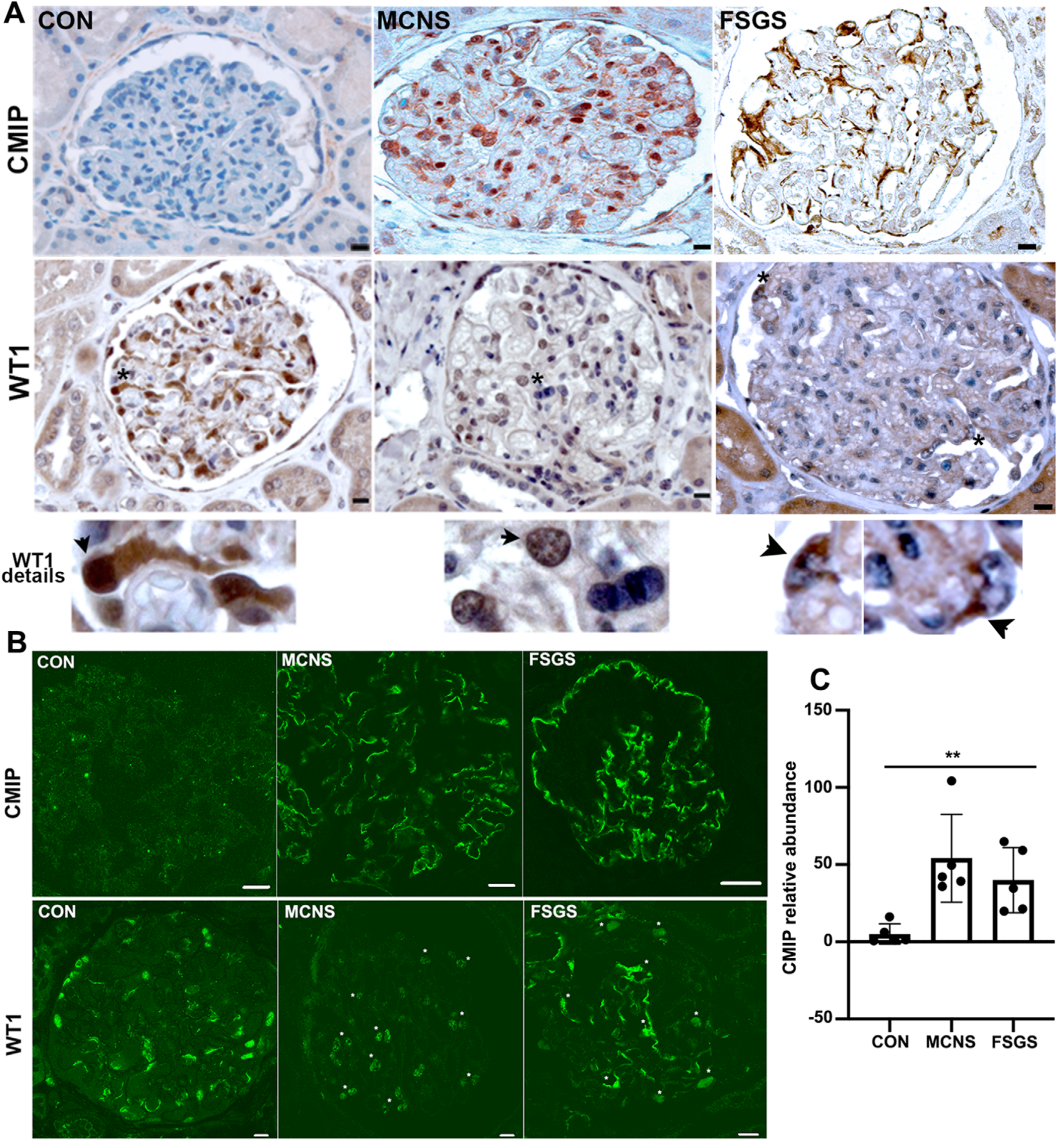
The abundance of WT1 decreases in the glomeruli of patients with MCNS and FSGS. (A) Representative immunohistochemical analysis of CMIP (upper level) and WT1 (lower level) in serial sections from control human kidney (CON, *n* = 5) and kidney biopsy specimens of patients with MCNS (*n* = 18) and FSGS (*n* = 5). Scale bar, 20 microns. Asterisks show the area enlarged in the glomeruli stained by WT1. Note that podocytes overexpressing CMIP exhibit a low abundance of WT1. (B) Confocal microscopy analysis of CMIP (top panel) and WT1 (lower panel) expression in healthy controls, MCNS and FSGS biopsy specimens. Asterisks show WT1 labeling. Scale bar, 20 microns. Note that the staining pattern of WT1 (a specific podocyte marker) showed a weak nuclear expression in FSGS with a more cytoplasmic distribution than in MCNS. (C) Quantitation of CMIP abundance in glomeruli of MCNS, FSGS, and controls (five biopsies each). All glomeruli from each biopsy were quantified, except those with advanced sclerosis. The abundance of CMIP was assessed by quantifying the specific glomerular fluorescence intensity (lining the capillary loops) in 3‐D stacks of images taken by confocal microscopy. The area of specific labeling (F) was normalized with respect to total glomerular area (S = labeled area/total area). The semi‐quantification (Q) of site‐specific fluorescent labeling (F) was determined as follows: Q = F x S, using Image J software. Data represent the means ± SD (**p = 0.0012; Kruskal‐Wallis test)

CMIP abundance was found increased in human and experimental models of idiopathic nephrotic syndrome (INS).^23^, ^37^, ^38^ Unexpectedly, we observed that the increase of CMIP abundance was consistently associated with a downregulation of WT1 in cell lines and in transgenic mice overexpressing CMIP. These observations led us to investigate whether CMIP influences the expression of WT1 in podocyte diseases. Immunohistochemical analysis of kidney biopsy specimens from patients with MCNS (*n* = 18) and FSGS (*n* = 5) revealed that glomeruli exhibited lower WT1 abundance compared to those of control human kidneys (Figure 1A). In FSGS, the expression of WT1 was reduced with an extranuclear distribution, which was more prominent compared to MCNS biopsies. Confocal microscopy analysis showed that CMIP expression was clearly induced in MCNS and FSGS whereas it was scarcely detected in healthy controls (Figures 1B and 1C). The expression of WT1 was reduced in MCNS with a speckled distribution or at the periphery of the nucleus. In FSGS biopsies, the expression pattern of WT1 showed diffuse distribution in rare nuclei while expression outside nuclei was more prominent than in MCNS.

### 3.2 CMIP interferes with NF‐kB‐mediated transcriptional activation of WT1

**FIGURE 2:**
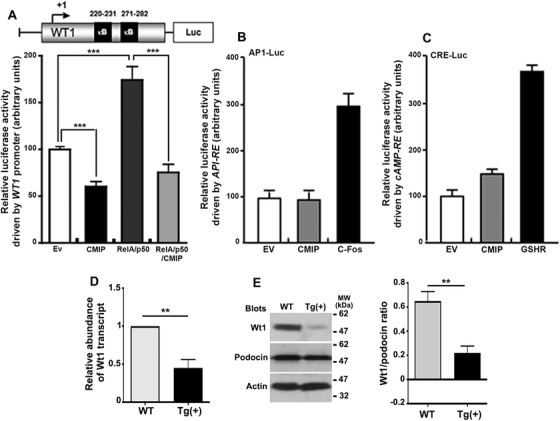
CMIP downregulates the NF‐kB‐mediated transcriptional activation of *WT1*, but does not influence the AP1 or CRE‐dependent transcriptional activities. (A) CMIP inhibits *WT1* promoter‐dependent luciferase activity. HEK cells were transiently co‐transfected with expression plasmids encoding CMIP or empty vector (Ev) and with/without NF‐kB p50/p65 subunits, and a *WT1* promoter‐Luc reporter plasmid construct. Promoter activity was measured 24 h following transfection, as relative luciferase activity (firefly luciferase/renilla luciferase). (B and C) Effect of CMIP on AP1 and CRE activation. HEK cells were co‐transfected with CMIP or empty vector (Ev), AP1‐luc, or CRE‐luc reporter plasmid. The Ghrelin receptor (GHSR) or c‐fos expression plasmid was used as a positive control. Results are representative of five independent experiments (mean ± SD, ***p < 0.001; Student's two‐tailed *t*‐test). (D) RT‐qPCR of *Wt1* transcript in 12‐week‐old wild‐type (Balb/c) and transgenic mice (*n* = 5 each). Quantification is measured as ‐fold induction relatively to control wild‐type (mean ± SD, **p = 0.0065; Mann–Whitney test). (E) immunoblotting of WT1 from glomerular extracts (mean ± SD, **p = 0.0058; Mann–Whitney test). These experiments were performed in 12‐week‐old wild‐type (Balb/c) and transgenic mice (*n* = 5 each)

The downregulation of WT1 in the presence of CMIP raises a possible mechanistic association. Although CMIP cannot be structurally considered as a transcription factor, we looked at whether it modulates *WT1* expression at the transcriptional level. It has been shown that NF‐kB binds to its responsive sites located on the human *WT1* promoter (positions 220–231 and 271–282) and induces a potent transactivation of *WT1*.^39^ Therefore, we measured luciferase activity in cell lysates from HEK cells cotransfected with *CMIP* expression vector and a human *WT1* promoter/luciferase reporter gene construct (pGLWTpH‐P) containing two NF‐kB regulatory sequences located at position 220–231 and 271–282 within the 5′ UTR.^29^ Luciferase activity driven by the *WT1* promoter was reduced by 40% relative to empty vector‐transfected cells (Figure 2A). We next studied the influence of CMIP on the NF‐kB‐mediated transcriptional activation of *WT1*. We measured luciferase activity 24 h after cotransfection of HEK cells with NF‐kB p50/RelA and the pGLWTpH‐P reporter gene construct, with or without a *CMIP* expression plasmid. NF‐kB induced a 75% increase in luciferase activity, relative to empty vector, which was abrogated in the presence of CMIP (Figure 2A, *p* < 0.001). By contrast, overproduction of CMIP did not affect luciferase activity driven by the AP1‐responsive element or cAMP‐responsive element (CRE) (Figures 2B and 2C). To investigate the relevance of these findings *in vivo*, we analyzed the expression of Wt1 in transgenic (Tg) mice, which selectively express human CMIP in podocytes.^23^ The amount of *Wt1* transcript in total glomerular RNA, as measured by quantitative RT‐PCR, was found to be reduced in 3‐month‐old Tg(+) mice when compared to wild‐type mice of the same age (Figure 2D). The ratio between (+KTS) and (‐KTS) spliced forms of the *Wt1* transcript, as measured by quantitative PCR in wild‐type mice and Tg(+) mice was not different (data not shown). The level of Wt1 protein in glomerular extracts, as revealed by Western blotting, was found decreased in Tg(+) mice (Figure 2E).

### 3.3 CMIP destabilizes WT1 protein

**FIGURE 3:**
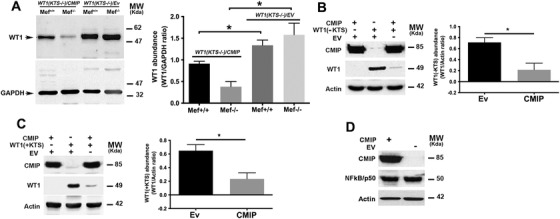
CMIP destabilizes WT1 (+/‐ KTS) at the post‐transcriptional level but does not affect the expression of the transcription factor NF‐kB p50. (A) Western blot analyses of WT1 expression in total cell lysates from wild‐type (Mef+/+) and RelA‐deficient mouse embryonic fibroblast (MEF‐/‐) transiently transfected with WT1(‐KTS) and with/without CMIP expression plasmids. The total amount of DNA was kept constant by replacing an expression plasmid by empty pcDNA vector. Note that in the absence of NF‐kB RelA, CMIP maintains its repressive effect on WT1 expression (mean ± SD, **p = 0.050; Mann–Whitney test). (B) Western blot analyses of WT1 expression in total cell lysates from HEK cells transiently transfected with CMIP, WT1(‐KTS) or both expression plasmids (mean ± SD, **p = 0.01; Mann–Whitney test). (C) Western blot analyses of WT1 expression in total cell lysates from HEK cells transiently transfected with CMIP, WT1(+KTS) or both expression plasmids. The empty vector was included for each single transfection (mean ± SD, **p = 0.01; Mann–Whitney test). (D) Western blot analyses of NF‐κB p50 following transfection of CMIP expression plasmids into HEK cells. Actin was used as internal control for the loading. Results are representative of three independent experiments. The band intensities were quantified using the Image J software and normalized to GAPDH or actin loading

Although CMIP inhibits NF‐kB‐mediated transcriptional activation of WT1, this mechanism does not seem to totally account for the dramatic decrease of WT1 abundance. To precisely determine the relative contribution of NF‐kB, we transiently cotransfected *CMIP* and *WT1* expression plasmids in wild‐type (MEF^+/+^) and RelA‐deficient (MEF^−/−^) mouse embryonic fibroblasts (MEF). In the absence of CMIP, WT1 abundance was not different between MEF^+/+^ and MEF^−/−^ (Figure 3A). On the other hand, WT1 abundance was reduced in CMIP‐overexpressing MEF with a major decrease in MEF^−/‐,^ suggesting that CMIP downregulates WT1 at transcriptional (through inhibition of NF‐kB) and post‐transcriptional level. Two main WT1 main isoforms are generated by alternative splicing through two splice donor sites in exon 9, leading to the insertion (WT1/+KTS) or deletion (WT1/‐KTS) of three aminoacids (lysine, threonine and serine: KTS) between zinc fingers 3 and 4 of the WT1 protein. While WT1/‐KTS is believed to act primarily as a transcriptional regulator, WT1/+KTS is involved in post‐transcriptional processing of RNA.^40^ We found that CMIP efficiently downregulated both isoforms (Figure 3A). The effect of CMIP on WT1 stability was not cell line‐dependent since it was also observed in HEK cells and specifically targets WT1 since overexpression of CMIP did not affect the abundance of endogenous NF‐kBp50 (Figures 3B–3D).

### 3.4 CMIP binds WT1 *in vivo*, displays E3 ligase activity, and targets WT1 to proteasome degradation

**FIGURE 4:**
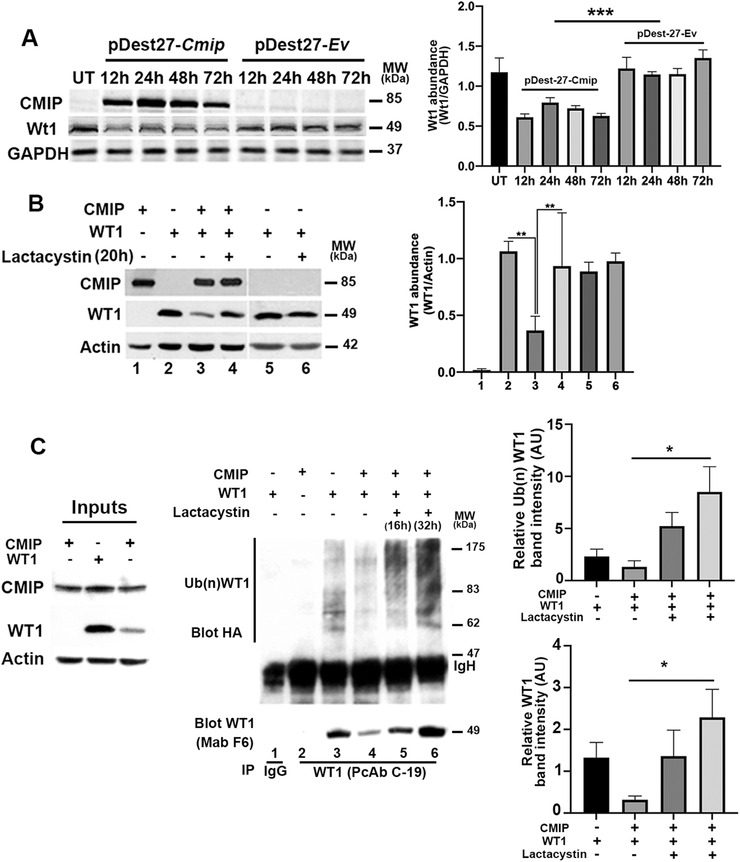
CMIP targets WT1 to the proteasome‐dependent degradation pathway, which is prevented by lactacystin. (A) Western blot analyses of Wt1 abundance in total cell lysates from M15 cells untransfected (UT) or transiently transfected with *Cmip* expression plasmid or control vector; Wt1 and Cmip were revealed by C‐19 antibody (sc‐192) and anti‐CMIP antibody, respectively. The band intensities were quantified using the Image J software and normalized to actin loading. (means ± SD, ***p = 0.0002; Mann‐Whitney test). (B) Western blot analyses of WT1 abundance in total cell lysates from HEK cells transiently transfected with *CMIP*, *WT1*(*‐KTS*) or both expression plasmids. Four hours post‐transfection, lactacystin was or not added to the medium culture for a period of 20 h. The band intensities were quantified using the Image J software and normalized to actin loading. (means ± SD, **p = 0.0079; Mann‐Whitney test). Results are representatives of five independent experiments. (C) Lactacystin inhibits degradation of ubiquitinated WT1. HEK cells were cotransfected with *CMIP*, *WT1*(*‐KTS*) and HA‐Ub, then treated or not with lactacystin for the indicated times. Protein lysates were immunoprecipitated with rabbit anti‐WT1 polyclonal antibody (C‐19), followed by immunoblotting of the eluates with rabbit anti‐HA polyclonal antibody or mouse anti‐Wt1 monoclonal antibody (F6). The relative intensity of ubiquitinated‐WT1 and WT1 protein bands were quantified using Image J software (means ± SD, Ub(n)WT1: *p = 0.03; WT1: (*p = 0.0112, one‐way ANOVA tests)

We next examined the effect of CMIP overexpression on Wt1 abundance in M15 cell line, which expresses a high level of Wt1,^31^ but does not express Cmip (Figure 4A). It has been established that human WT1 (accession number: NP_077744.3) and mouse counterpart (accession number: NP_659032.2) proteins share 98% of homology.^41^ The Figure 4A shows that Wt1 abundance fell by 50% at 12 h after transfection of M15 cells with Cmip (mouse Cmip). Because transiently transfected plasmids do not generally integrate into the genome of recipient cells,^42^ these results suggest that Cmip destabilizes the Wt1 protein independently of its indirect transcriptional effect.

The proteasome represents one of the main degradation pathways of regulatory proteins such as WT1.^43^ To determine whether CMIP affects the stability of WT1, we cotransfected WT1 and CMIP expression plasmids into HEK cells and 4 h later, the cells were incubated with lactacystin, a potent inhibitor of the proteasome, which does not interfere with any other proteases or with lysosomal protein degradation.^44^ The abundance of WT1 protein was reduced (∼ 60%) in the presence of CMIP, but coincubation with lactacystin inhibited WT1 decay (Figure 4B), suggesting that CMIP targets WT1 to proteasome‐mediated degradation. By contrast, lactacystin has no effect on WT1 abundance in the absence of CMIP. To assess whether the degradation of WT1 by the proteasome requires its polyubiquitination beforehand, HEK cells were cotransfected with CMIP and WT1 along with a construct expressing hemagglutinin‐tagged ubiquitin (HA‐Ub), under conditions identical to those used above, and WT1 was immunoprecipitated from protein lysates. Immunoblotting of the eluates with anti‐HA revealed a smear corresponding to high molecular weight species (∼ 60–190 kDa) of ubiquitin molecules covalently linked to WT1 (Figure 4C). In the presence of CMIP, WT1 was mostly degraded presumably by extensive polyubiquitination, so that little WT1‐ubiquitin was detectable. On the other hand, inhibition of the proteasome induced an accumulation of ubiquitinated WT1 with a shift towards high molecular weight species (Figure 4C).

**FIGURE 5:**
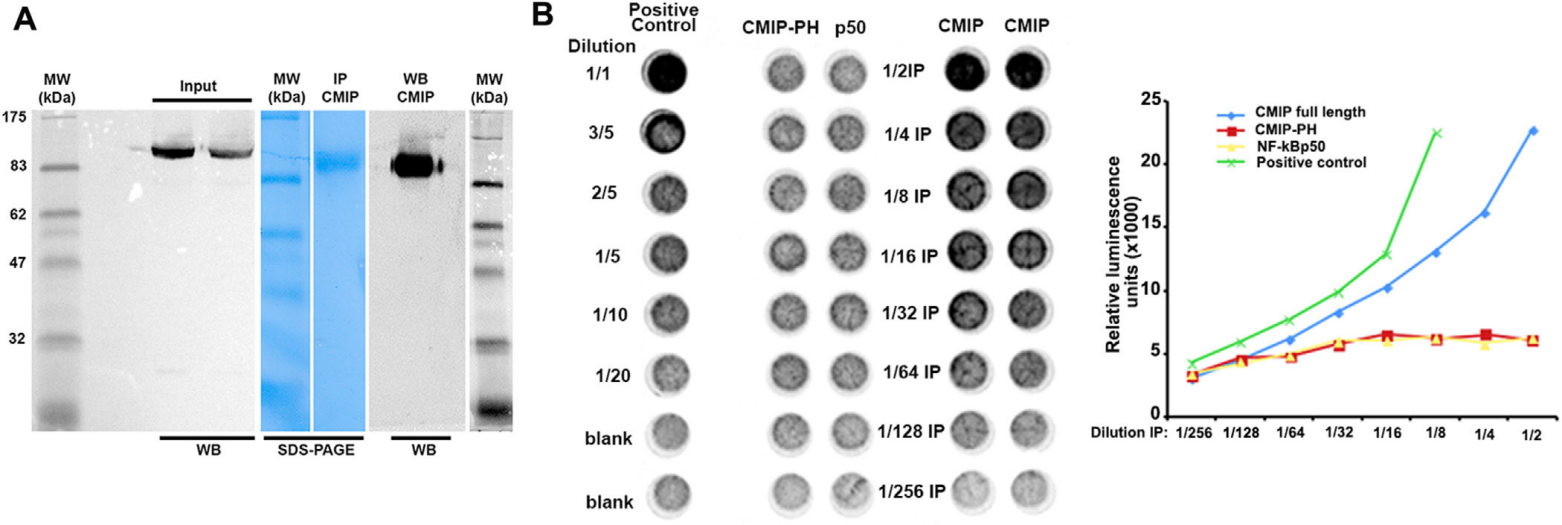
CMIP generates polyubiquitin chains in the presence of ubiquitins and E1‐E2 enzymes, suggesting an E3 ligase activity. (A) HEK cells were transfected with whole CMIP (pDEST27‐CMIP), CMIP‐PH (pDEST27‐CMIP‐PH) or NF‐kBp50 expression plasmids. The quality of whole CMIP preparations was tested by Western‐blot (WB) before (input) and after immunoprepcipitation (IP) using GST‐agarose beads by SDS‐PAGE and WB. (B) CMIP was assessed for an E3 ligase activity, as detailed in Materials and Methods. The controls include truncated CMIP (Cmip‐PH) and NF‐kB p50 subunit. The generation of polyubiquitin chains was detected by enhanced chemiluminescent (ECL) reaction. Values measured in duplicate wells minus those obtained in the blank control in each dilution were represented in the graph (right of the panel). Results are representative of three independent experiments

**FIGURE 6:**
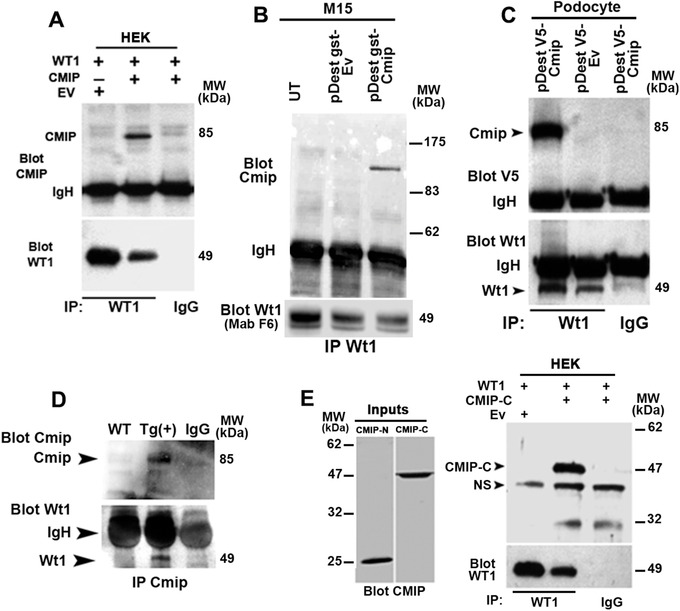
CMIP interacts with WT1 *in vitro* and *in vivo*. (A) Immunoprecipitation of WT1 from total cell lysates of HEK cells cotransfected with CMIP, WT1, or both. Results are representative of six independent experiments. (B) M15 cells (which express endogenous WT1) were cotransfected with Cmip expression plasmid or empty vector (Ev). Protein lysates were immunoprecipitated with anti‐WT1 (polyclonal C19), and eluates were probed with anti‐Cmip or anti‐WT1 (Mab F6). (C) Immunoprecipitation of WT1 from extracts of a podocyte cell line transfected with V5‐tagged Cmip, followed by immunoblotting of the eluates with anti‐WT1 or anti‐V5‐tag antibodies (V5 is a small sequence encoding 9–14 amino‐acids used for detection of tagged target protein). (D) Immunoprecipitation of Cmip from glomerular extracts of wild‐type and Tg(+) mice followed by immunoblotting of the eluates with anti‐WT1 and anti‐CMIP antibodies. (E) CMIP interacts with WT1 via its C‐terminal region containing the LRR domain. The inputs of truncated forms (CMIP‐N containing the PH domain and CMIP‐C containing the LRR domain are indicated in E, left. The right of the panel shows immunoprecipitation of WT1 from total extracts of HEK cells cotransfected with expression plasmids encoding WT1 or truncated forms of CMIP (NS denotes nonspecific binding)

**FIGURE 7:**
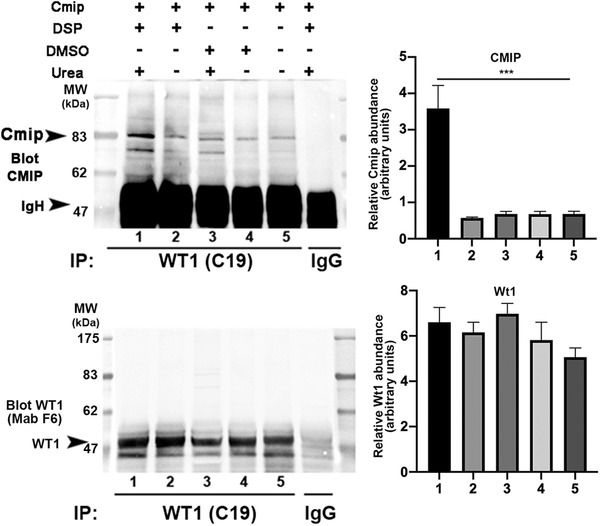
CMIP interacts with WT1. *In vivo* cross‐linking. M15 cells were transfected with mouse Cmip and treated for 20 min with 0.5 mM cross‐linker DSP or vehicle (DMSO). Cell protein extracts were prepared in lysis buffer with or without 8 M urea and immunoprecipitated with rabbit anti‐Wt1 antibody. The eluates were probed with rabbit anti‐Cmip antibody or mouse anti‐Wt1 monoclonal antibody (F6). Note that Cmip interacts directly with endogenous Wt1. The relative intensity of Cmip and Wt1 protein bands were quantified using Image J software. Data are representative of three independent experiments (Cmip, means ± SD, ***p < 0.001, one‐way ANOVA tests)

These results led us to ask whether CMIP could have an E3 ligase activity that drives ubiquitinylation of WT1. To test this hypothesis, CMIP was purified by immunoprecipitation from HEK cells overexpressing CMIP (Figure 5A), and its ability to generate polyubiquitin chains in the presence of ubiquitin and E1‐E2 enzymes but without any E3 ligase was determined. We found that CMIP stimulated the formation of polyubiquitin chains in a dose dependent manner (Figure 5B), suggesting an intrinsic ubiquitin E3 ligase activity. By contrast, E3 ligase activity was not detected when NF‐kB p50 or a truncated CMIP form containing only the PH domain (CMIP‐PH) was added to the ligation reaction instead of CMIP. To determine whether this process requires the interaction of WT1 with CMIP, several IP experiments were performed. HEK cells were transiently cotransfected with CMIP and WT1/‐KTS expression plasmids, then whole cell lysates were immunoprecipitated with an anti‐WT1 antibody and the eluates probed with anti‐CMIP antibody. We found that CMIP was coimmunoprecipitated with WT1 (Figure 6A). The interaction of CMIP with endogenous Wt1 was confirmed in M15 cell line (Figure 6B) and in mouse podocyte cell lines transfected with CMIP (Figure 6C). In addition, we showed that Cmip co‐immunoprecipitated with Wt1 *in vivo* in Tg mice (Figure 6D). To identify the region that binds to WT1, we truncated CMIP and performed similar cotransfection experiments in HEK cells. Only the C‐terminal segment containing the LRR domain (CMIP‐C) was consistently found to interact with WT1 (Figure 6E), whereas the PH domain‐containing region (CMIP‐N) did not coimmunoprecipitate with WT1 (data not shown). To assess whether CMIP interacts directly with WT1 *in vivo*, we generated covalent linkages between the proteins in M15 cell line by treating them with DSP, a thiol‐cleavable homobifunctional ester causing linkages principally through primary amines.^18^, ^45^ The urea was included in the lysis buffer to suppress any indirect or nonspecific interaction. Immunnoblot analysis shows that CMIP was clearly detected in the eluates from urea‐treated cross‐linked M15 protein lysates immunoprecipitated with WT1 antibody (Figure 7). Taken together, these results suggest that CMIP interacts directly with WT1 through its LRR domain and targets it for proteasomal‐mediated degradation.

### 3.5 RNAi knockdown of Cmip restores the expression of WT1 in LPS‐treated mice

**FIGURE 8:**
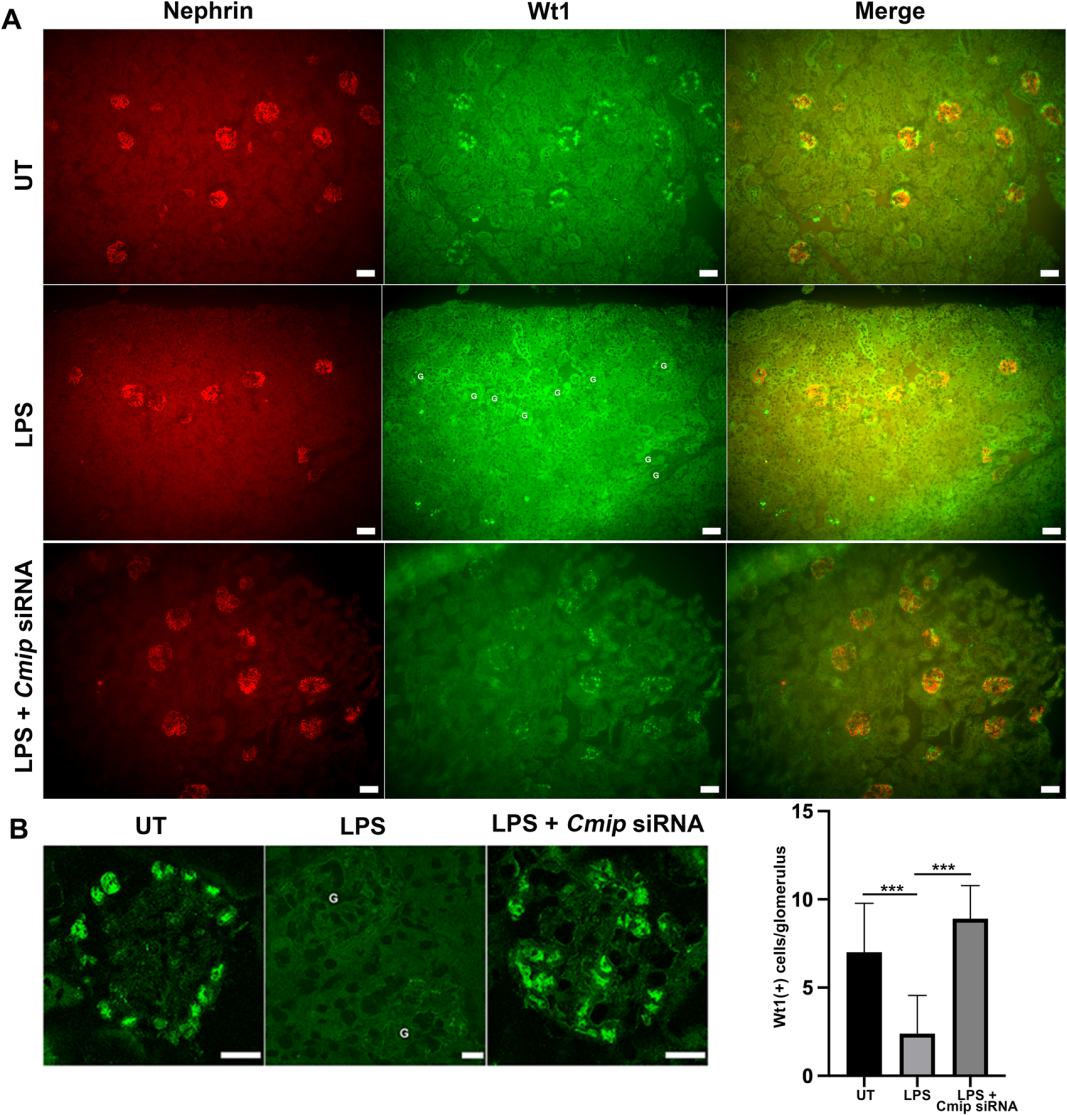
Knockdown of Cmip prevents the downregulation of Wt1. (A) Representative double‐immunofluorescence analysis with anti‐WT1 (green) and anti‐nephrin (red) in kidney sections in untreated (UT) mice (upper panel), LPS‐treated mice (middle panel) and LPS/Cmip siRNA‐treated mice (lower panel). Note that the expression of WT1 in glomeruli (G) is preserved in LPS/Cmip siRNA‐treated mice, as compared with LPS alone. Scale bar, 20 microns. (B) Upper panel, confocal microscopy analysis of kidney sections stained with anti‐WT1 antibody in untreated (UT) mice, LPS‐treated mice and LPS/Cmip siRNA‐treated mice. Note that the expression of Wt1 is preserved in mice treated with LPS and Cmip‐siRNA, as compared with LPS alone. Scale bar, 10 microns; lower panel, mean number of WT1‐positive cells by glomerulus. Data are means ± SD. The abundance of WT1 is lower in LPS‐treated mice (*n* = 5 mice), than in LPS/Cmip siRNA‐treated mice (*n* = 5 mice) (30 glomeruli were analyzed per mouse, ***p < 0.001, Mann‐Whitney test)

**FIGURE 9:**
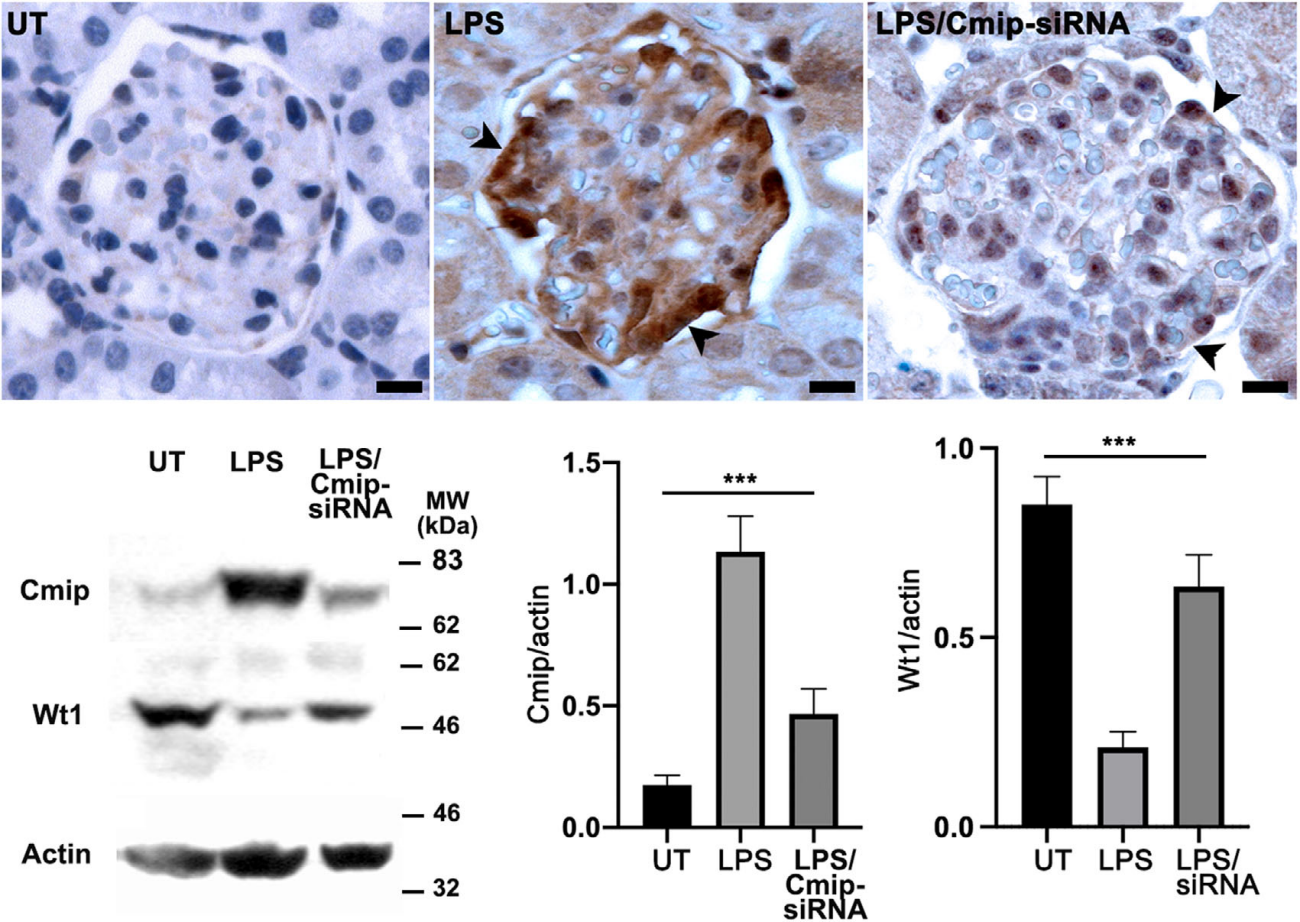
Cmip siRNA prevents LPS‐mediated Cmip upregulation. Quantitative expression of Cmip and Wt1. (A) Representative immunohistochemistry analysis of Cmip in kidney sections in untreated (left), LPS‐treated (middle panel) and LPS/Cmip siRNA‐treated mice (right panel). Note that Cmip abundance in glomeruli is increased in mice treated with LPS and decreased after specific siRNA. Scale bars, 10 microns. (B) Quantitative abundance of Cmip and Wt1 in glomeruli. Glomerular protein extracts were prepared from each group of mice (*n* = 5) and tested by Western blot for the expression of Cmip and Wt1. Band intensities were quantified using the Image J software and normalized to actin loading. (means ± SD, ***p < 0.001, ordinary one‐way ANOVA test)

We observed that LPS‐treated mice exhibited an upregulation of (endogenous) Cmip in podocytes concomitant with induction of proteinuria.^23^ In agreement with other authors,^20^ we showed that LPS induced a downregulation of Wt1 (Figure 8A). To address the question whether the repression of Wt1 by LPS could be prevented by Cmip knockdown, we performed *in vivo* RNA interference targeting Cmip in Wt mice. Confocal immunofluorescence analysis showed that abundance of WT1 was higher in LPS/Cmip siRNA‐treated mice, while it was decreased in LPS‐mice, when compared to untreated (UT) mice (Figure 8B). Alongside, we examined the abundance of Cmip and Wt1 by immunohistochemistry and quantitative Western blots. While Cmip was scarcely detected in UT mice, its expression was strongly induced in mice receiving LPS alone (Figure 9). In contrast, Cmip abundance fell in mice injected with Cmip siRNA and LPS. These results suggest that Cmip exerts a potent repressor effect on WT1 expression, which might contribute to podocyte disorders.

## 4 DISCUSSION

In the adult glomeruli, WT1 expression is restricted to podocytes and is essential for the functional integrity of the glomerular filtration barrier. The potential alterations of WT1 in acquired glomerular diseases and their functional consequences are incompletely understood. Based on the present study, we come to the following conclusions: (1) Overproduction of CMIP in acquired INS is associated with a downregulation of WT1 according to two mechanisms: (i) CMIP inhibits NF‐kB‐mediated transcriptional activation of WT1; (ii) CMIP interacts directly with WT1 and displays E3 ligase activity, which promotes WT1 degradation through proteasome pathway; (2) silencing endogenous Cmip expression with RNAi prevents the downregulation of WT1 in LPS‐treated mice.

E3 Ub ligases act as scaffold proteins, which facilitate the transfer of ubiquitins from the E2‐conjuguating enzyme to a lysine residue in the target protein, by forming an intermediate complex with the E2 and the target.^46^ E3 Ub ligases determine the enzymatic specificity by providing target specificity. They are classified into two large families: RING‐type E3 ligases and HECT‐type E3 ligases.^47^ Although CMIP contains none of the typical domains previously described in other E3 Ub ligases, the following evidences suggest that it might be a bona fide E3 Ub ligase for WT1: (1) The ubiquitination of WT1 is increased in the presence of CMIP. (2) The reduction of WT1 abundance is prevented by lactacystin, and^3^ CMIP binds directly WT1 in vivo and displays an E3 Ub ligase activity. Furthermore, other atypical E3 ligases have been reported.^48^, ^49^

CMIP inhibits NF‐kB‐mediated transactivation of the *WT1* gene, which might account for the downregulation of *WT1* transcript as detected in transgenic mice. We have previously shown that CMIP interacts with NF‐kB/RelA through its LRR domain and inhibits its nuclear translocation, thus preventing the transcriptional activation of NF‐kB target genes such as WT1.^24^ Interestingly sorafenib, a tyrosine kinase inhibitor, induces an upregulation of CMIP in podocyte, along with cytoplasmic sequestration of RelA.^27^ We show here an additional, post‐transcriptional, repression of WT1 in that CMIP promotes the degradation of WT1 by the proteasome through its E3 ligase activity. These results suggest that the sustained upregulation of CMIP in podocytes and the subsequent reduction in WT1 expression levels could dramatically affect the function and survival of these cells, leading to glomerulosclerosis, as observed in primary FSGS.^26^ Although downregulation of WT1 is usually attributed to podocyte loss, several arguments suggest that WT1 may be reduced in situations that are not necessarily accompanied by a podocyte depletion: (1) The abundance of WT1 was reduced in a reversible fashion in experimental nephrotic proteinuria induced by LPS^20^ and Adriamycin.^21^ (2) Podocyte depletion does not occur in MCNS disease except in chronic steroid resistant forms. (3) In Cmip transgenic mice, WT1 is diminished as early as 8–12 weeks of age even though no histological alterations or podocyturia were clearly evidenced at this time (data not shown). (4) Podocyte depletion is a late event and does not account for the dynamic alterations mediated by Cmip on WT1 activity.

A negative reciprocal regulation, as observed between Cmip and WT1, has already been reported in other biological systems where molecules are involved in opposite signaling pathways, as those involved in cell survival and death or inflammation and wound healing. Such examples include the reciprocal regulation between PDK1 and ASK1^50^ and between cox2 and p53.^51^ We have also recently reported a negative crosstalk between CMIP and NF‐kB‐RelA, suggesting that this phenomenon is more common than previously expected.^27^ Interestingly, a recent study showed that the downregulation of WT1 by miR‐193a promotes an increase in CMIP abundance and inhibition of NF‐kb/RelA activity leading to podocyte apoptosis.^52^

Several proteins that interact with WT1 modulate its transcriptional activity. The tumor suppressor factor p53 prevents the activation, but facilitates the repressive function of WT1.^53^ Similarly, Par‐4, a leucine zipper domain‐containing protein interacts with WT1 and inhibits WT1‐mediated transcriptional activation while enhancing the ability of WT1 to repress transcription.^54^ Ciao‐1, a novel member of the WD40 family, has been shown to bind WT1 and inhibit its transcriptional activation..^55^ In contrast, CMIP differs from known WT1‐interacting proteins in several respects: (1) CMIP is expressed at low level under physiological conditions but is induced in some pathological situations. (2) To our knowledge, CMIP is the first molecule that binds WT1 and targets it to proteasome degradation through its E3 ligase activity.

A significant reduction in WT1 expression was found in MCNS and FSGS, the main causes of podocytopathies.^56^ In both cases, increased CMIP abundance is demonstrated *in vivo* but the lesser sensitivity to therapy suggests that additional mechanisms are involved in FSGS. In the absence of response to therapy, the persistent expression of CMIP may contribute to the development of podocyte damages and, belatedly, of glomerulosclerosis. This observation raises the hypothesis of whether structural modifications in the *CMIP* gene, which spans 270 Mb, including epigenetic changes could be associated with a defect in its regulation in a pathological context and confers resistance to therapy. The pathophysiological link between MCNS and primary FSGS remains widely debated. Overlap of histological lesions may occur during the course of MCNS, while FSGS can fully respond to steroid therapy, suggesting a continuum between the two entities. However, as long as the pathogenesis of primary INS remains unclear, ascribing any link is speculative.

We have previously shown that CMIP interacts with the Src kinase Fyn and inhibits phosphorylation of nephrin and N‐WASP, leading to cytoskeleton disorganization.^23^ The relative contribution of the effects of CMIP in respect of nephrin signaling and WT decay on the pathophysiology of podocyte diseases remains to be clarified.

## 5 CONCLUSION

WT1 is constitutively expressed in glomeruli and is essential to ensure podocyte structure, function, and maintenance through the activation of numerous specific genes. While in human pathology genetic inactivation of WT1 leads to podocyte apoptosis and glomerulosclerosis, little is known how WT1 is deregulated in acquired glomerular diseases. Here, we provide experimental evidence that CMIP can destabilize WT1 according to two mechanisms: CMIP inhibits NF‐kB‐mediated transcriptional activation of WT1 and induces its degradation through the ubiquitin‐proteasome pathway. Our results suggest CMIP as a potential therapeutic target.

## CONFLICT OF INTEREST

The authors declare that they do not have any competing financial, personal, or professional interest.

### AUTHOR CONTRIBUTIONS

Shao‐Yu Zhang, Qingfeng Fan, Anissa Moktefi, Virginie Ory, and Andre Pawlak performed the experiments. Vincent Audard collected clinical data and tissue samples. Vincent Audard, Mario Ollero, Carole Henique, and Dil Sahali wrote the paper. Dil Sahali supervised the project. All authors discussed the results and participated in writing the paper.
